# Collection and preprocessing of fine needle aspirate patient samples for single cell profiling and data analysis

**DOI:** 10.1016/j.xpro.2021.100581

**Published:** 2021-06-05

**Authors:** Aizhan Tastanova, Egle Ramelyte, Zsolt Balázs, Ulrike Menzel, Christian Beisel, Michael Krauthammer, Reinhard Dummer, Mitchell Paul Levesque

**Affiliations:** 1 Dermatology Department, University Hospital Zurich and Medical Faculty, University of Zurich, 8091 Zurich, Switzerland; 2 Department of Quantitative Biomedicine, University of Zurich, 8057 Zurich, Switzerland; 3 Biomedical Informatics, University Hospital of Zurich, 8057 Zurich, Switzerland; 4 Department of Biosystems Science and Engineering, ETH Zurich, 4058 Basel, Switzerland

**Keywords:** Bioinformatics, Cell isolation, Single Cell, Clinical Protocol, RNAseq

## Abstract

High cell viability and recovered cell concentration are typical quality control requirements for single-cell processing and quality data. This protocol describes procedures for sampling, live-cell biobanking, preprocessing for single-cell RNA sequencing, and analysis of fine-needle aspiration (FNA) samples of the skin. The minimally invasive nature of FNA collection is more accepted by patients and allows for frequent longitudinal sampling, resulting in high-quality single-cell sequencing data that capture cellular heterogeneity in clinical samples.

## Highlights

• Protocol for fine-needle aspirate collection from human skin lesions
• Details for pre-processing of samples for single-cell RNA sequencing
• RNA-seq data analysis with immune repertoire analysis and quality control

## Graphical abstract

**Figure:**
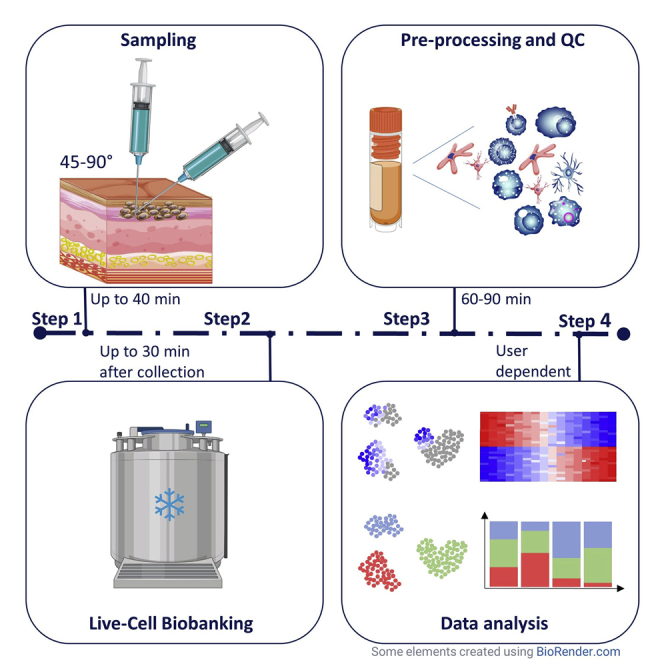

## Before you begin

The protocol below outlines preparation steps for fine needle aspirate collection (FNA) and its preprocessing for single cell RNA sequencing on cutaneous cancer lesions. We have performed the FNA sampling on cutaneous B cell lymphoma and cutaneous melanoma metastases.

### Preparation for FNA procedure and biobanking of clinical samples

**Timing: 20 min**

1. Prepare the syringe holder (e.g., Pistomed, MED1000522, medesign, Dietramszell - Linden, Germany) suiting syringe (e.g., 20 mL) and needle (e.g., G23)
2. Prepare disinfecting wipes and a bandage
3. Prepare a 1 mL cryovial with slow freezing medium (FBS 90%, DMSO 10%) and place it on ice
4. Pre-cool the cryo container (CoolCell LX, BioCision, BCS-405G, Corning, Inc.).

***Alternatives:*** Mr. Frosty cooling container (Cat.#5100-0001, NALGENE^TM^ Cryo).

### Preparation for preprocessing of fresh or cryopreserved FNA samples for single-cell droplet generation

**Timing: 20 min**

5. Warm the water bath to 37°C (needed to quickly thaw if FNA samples were cryopreserved)
6. Prepare cold PBS (Ca^2+^/Mg^2+^ free, Gibco, Cat.#10010-015) containing 0.04% BSA (filter sterilized), keep on ice
7. Label 5 mL Eppendorf’s (Eppendorf, Cat.#0030122313) or 15 mL centrifuge tubes (TPP, Cat.#91015) with sample names/IDs
8. Prepare and label FACS tubes with blue filter cap (filter size 35 μM Cat.#352235, Falcon)
9. Prepare recommended medium for dead cell removal: PBS (Ca^2+^/Mg^2+^ free, Gibco, Cat.#10010-015) with 1% FBS (Cat.#S006420E01, Biowest) and 1 mM CaCl_2_ (Cat.# 746495, Sigma Aldrich) - for low viability samples
10. Prepare red blood cell lysis (Cat.#11814389001, ROCHE) for samples having red blood cells, important to remove them from downstream single cell processing.

***Optional:*** For convenience one may prepare a dry ice and keep the cryopreserved samples nearby until ready for processing

## Key resources table

**Table:** 

REAGENT or RESOURCE	SOURCE	IDENTIFIER
**Biological samples**		

Human primary cutaneous lymphoma—fine-needle aspirates	Dermatology Department, University Hospital Zurich	NA
Human metastatic melanoma—fine-needle aspirates	Dermatology Department, University Hospital Zurich	NA

**Chemicals, peptides, and recombinant proteins**		

DMSO	Sigma	Cat.#102148154
FBS	Biowest	Cat.#S006420E01
BSA (bovine serum albumin)	Sigma-Aldrich	Cat.#A7906
CaCl_2_	Sigma Aldrich	Cat.#746495

**Critical commercial assays**		

Chromium Single Cell V(D)J Reagent Kits (v1 Chemistry)	10x Genomics	PN-1000006 PN-1000020 PN-1000005 PN-1000016 PN-120236 PN-120262
Luna-FL^TM^ Dual Fluorescence Cell counter	Logos Biosystems Inc.	Cat.#L1001
PhotonSlides (ultra-low fluorescence counting slides)	Logos Biosystems Inc.	Cat.#L12005
Acridine orange propidium iodide (AOPI) stain	Logos Biosystems Inc.	Cat.#F23001
EasySep^TM^ Magnet	STEMCELL Technologies	Cat.#18000
Annexin Dead Cell Removal Kit	STEMCELL Technologies	Cat.#17899
Red Blood Cell Lysis Buffer	Roche	Cat.#11814389001
High Sensitivity DNA Kit	Agilent Technologies	Cat.#5067-4626
Agilent 2100 Bioanalyzer	Agilent Technologies	System no. G1030AX

**Software and algorithms**		

ScanScope CS	Aperio	http://scanscope.com/
Seurat	Satija Lab	https://satijalab.org/seurat/index.html
SingleR	Dvir Aran, Aaron Lun et al.	https://bioconductor.org/packages/release/bioc/html/SingleR.html
CellRanger	10x Genomics	https://support.10xgenomics.com/single-cell-gene-expression/software/pipelines/latest/what-is-cell-ranger
Scripts used for the T-VEC lymphoma study	Krauthammer Lab	https://github.com/uzh-dqbm-cmi/lymphoma_tvec_study

**Other**		

35-μm Cell strainers (blue capped FACS tubes)	Falcon	Cat.#352235
Eppendorf Tubes® 5.0 mL with Screw Cap	Eppendorf	Cat.#0030122313
Pistomed (A syringe holder)	Medesign	Cat.#MED1000522
Corning® CoolCell™ LX Cell Freezing Container	Merck	Cat.#CLS432001-1EA
15 mL Centrifuge tubes	TPP	Cat.#91015
Steriflip, 0.22 μM filter	Milipore	Cat.#SCGP00525

## Materials and equipment

**Table:** Washing and resuspension buffer (PBS with 0.04% BSA )

Reagent	Final concentration	Amount
BSA	1%	20 mL
PBS (Ca^2+^/Mg^2+^ free)	n/a	480 mL
**Total**	n/a	500 mL

Prepare 1% BSA by dissolving 1 g of BSA (Cat.#A7906, Sigma-Aldrich) in 100 mL of sterile PBS (Ca^2+^/Mg^2+^ free, Gibco, Cat.#10010-015) and filter through 0.22 μM filter (Steriflip, Milipore, Cat.#SCGP00525). Store for up to 1 month at 4°C, check for visible contaminations.

**Table:** Recommended medium for dead cell removal

Reagent	Final concentration	Amount
FBS	2%	200 μL
CaCl_2_	1 mM	200 μL
PBS (Ca^2+^/Mg^2+^ free)	n/a	9.6 mL
Total	n/a	10 mL

Always prepare fresh and filter the final solution with 0.22 μM filter (Steriflip, Milipore, Cat.#SCGP00525).

**Table:** Cryopreservation medium (also FNA collection medium)

Reagent	Final concentration	Amount
FCS	90%	900 μL
DMSO	10%	100 μL
Total	n/a	1 mL

Per sample preparation, prepare fresh, filter the final solution with 0.22 μM filter (Steriflip, Milipore Cat.#SCGP00525), store for a short time, up to one week at 4°C.

## Step-by-step method details

This section describes how to collect FNA samples from cutaneous cancers and cryopreserve using a slow-freezing procedure, as well as preprocess the cryopreserved or freshly sampled FNA sample for single cell RNA-seq processing and single-cell data analysis tools.

### FNA sampling

**Timing: 20 min**

FNA is a minimally invasive procedure that allows repetitive sample collection from the same lesion. This enables the profiling of changes in cell composition over time, e.g., before and during the therapy.

1. FNA procedure
  a. Disinfect the surface of the lesion with ethanol pads
  b. Fix the skin lesion between the thumb and index finger of the non-dominant hand to stabilize and prevent slipping of the lesion and accidental pre-mature syringe withdrawal (Figure 1)

**Figure 1:**
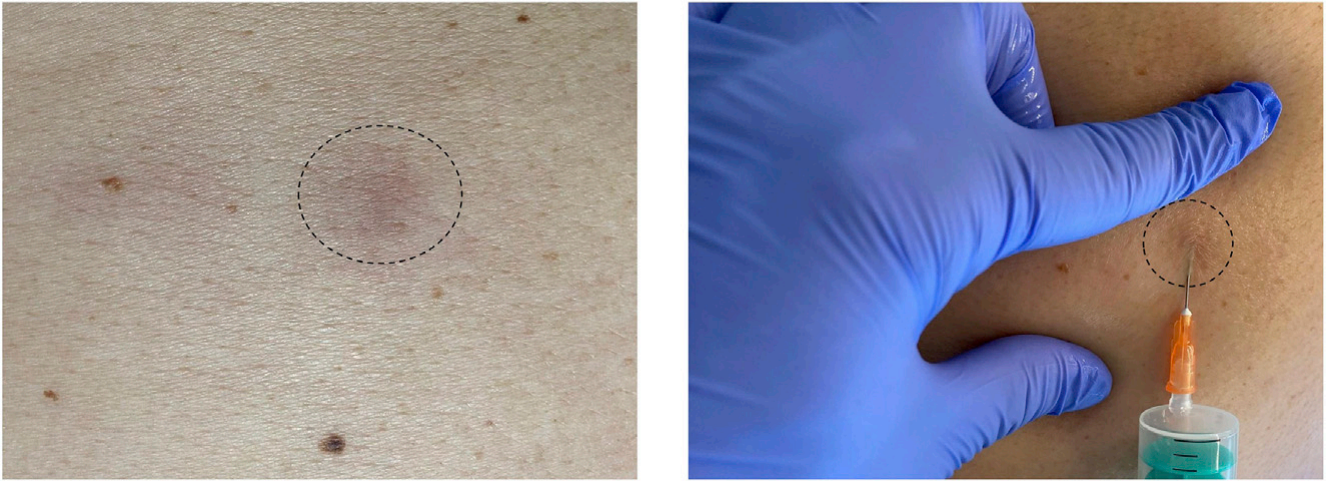
Representative image of a skin lesion (on the left) and the lesion fixation followed by insertion of a needle (on the right)
  c. At the edge of the lesion, aiming toward the center, insert the needle on a syringe (empty) fixed in the syringe holder (Pistomed) at approx. 90° angle in bigger tumors and ca 45° angle in superficial lesions, such as plaques (Figure 1). In case of accidental piercing through the lesion, pull the syringe back to keep the needle tip within the lesion proceed to the next step
  d. Pull the plunger to apply negative pressure once the lesion is penetrated. If the lesion is necrotic and you cannot create negative pressure, move the syringe within the lesion until it reaches a solid part and you feel resistance (creation of negative pressure) while pulling the plunger
  e. To sample as many parts of the lesion as possible, move the syringe in and out within the lesion in a fan-shaped manner for at least 10 times without taking the needle out of the lesion
  f. Once tissue material (reddish or yellowish, depending on the tumor type and erythrocyte content) appears in the syringe hub, release the plunger to remove negative pressure and slowly withdraw the needle from the lesion. If the syringe hub does not fill, move the syringe to other areas of the lesion or troubleshoot for needle clogging (see below), if the whole lesion area was penetrated
  g. Put a bandage on the needle insertion site
2. Transferring the sample to the slow-freezing medium
  a. Aspirate the slow freezing medium to the syringe, which contains the collected sample and put it back to the cryovial
  b. Keep the needle in the fluid and move the plunger in and out of the syringe to suspend the remaining cells in the syringe hub until most cells are transferred
  c. Close the cryovial and keep it on ice until biobanking.

***Alternatives:*** FNA samples can be processed fresh. When planning to process the samples freshly without cryopreservation, collect the samples in 1 mL 100% FCS. After collection, transfer into a 5 mL Eppendorf or 15 mL centrifuge tubes and top up with 4 mL or 9 mL of any medium (RPMI or DMEM), respectively. Keep on ice and process as soon as possible, we have processed freshly collected FNAs within 2 h after collection.

### FNA live-cell biobanking

**Timing: 30 min**

3. Keep the collected FNA sample on ice at all times. Place the cryovial with the FNA sample in pre-chilled Corning® CoolCell® Containers (432001) and put into −80° fridges within 30 min after collection.
4. Leave the Corning® CoolCell® Containers at −80° for at least 4 h and after the sample is fully frozen, transfer the samples to liquid nitrogen for prolonged storage.

**Pause point:** After the FNA sample is cryopreserved, it could be stored until convenient for processing. The samples could be stored in liquid nitrogen for at least 2 years. We recommend collecting at least eight samples to process simultaneously.

### Preprocessing FNA samples for single-cell droplet generation

**Timing: 60–90 min**

**Figure 2:**
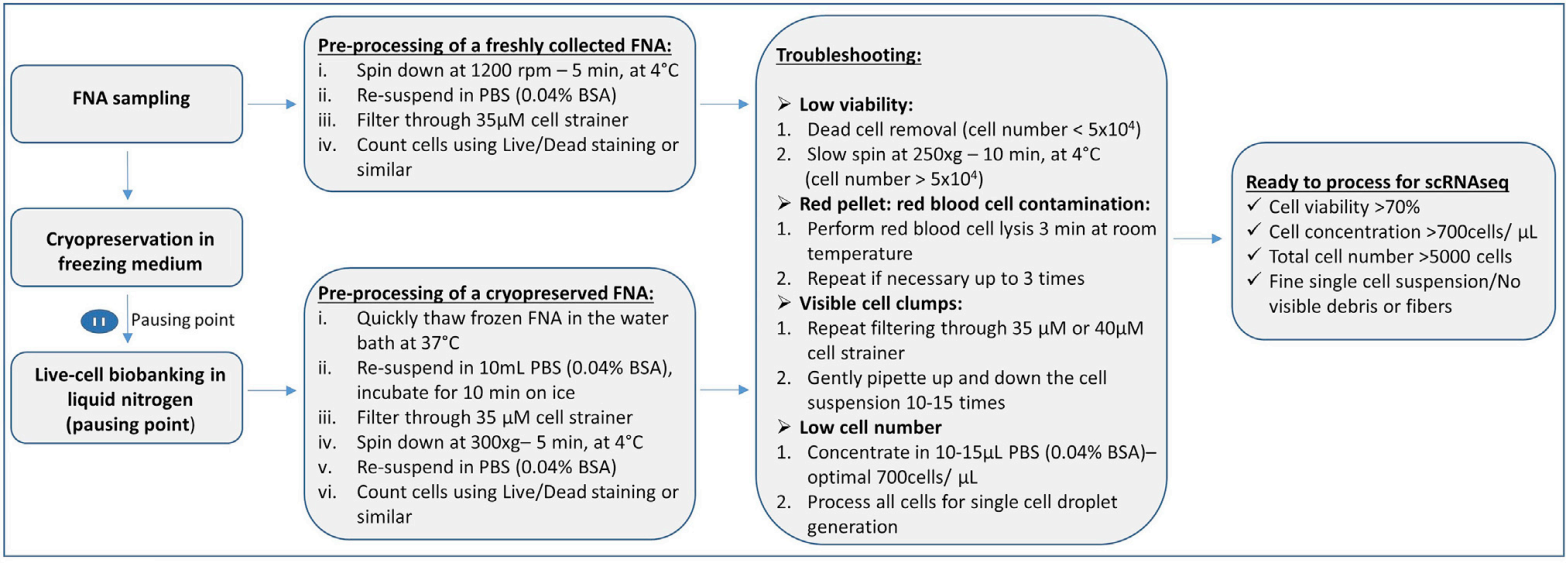
Preprocessing of freshly collected and live-cell biobanked FNA samples Main steps and troubleshooting are outlined.

Within this step, a cryopreserved FNA sample is thawed, assessed for quality and preprocessed for single cell droplet generation (Figure 2).

**Figure 3:**
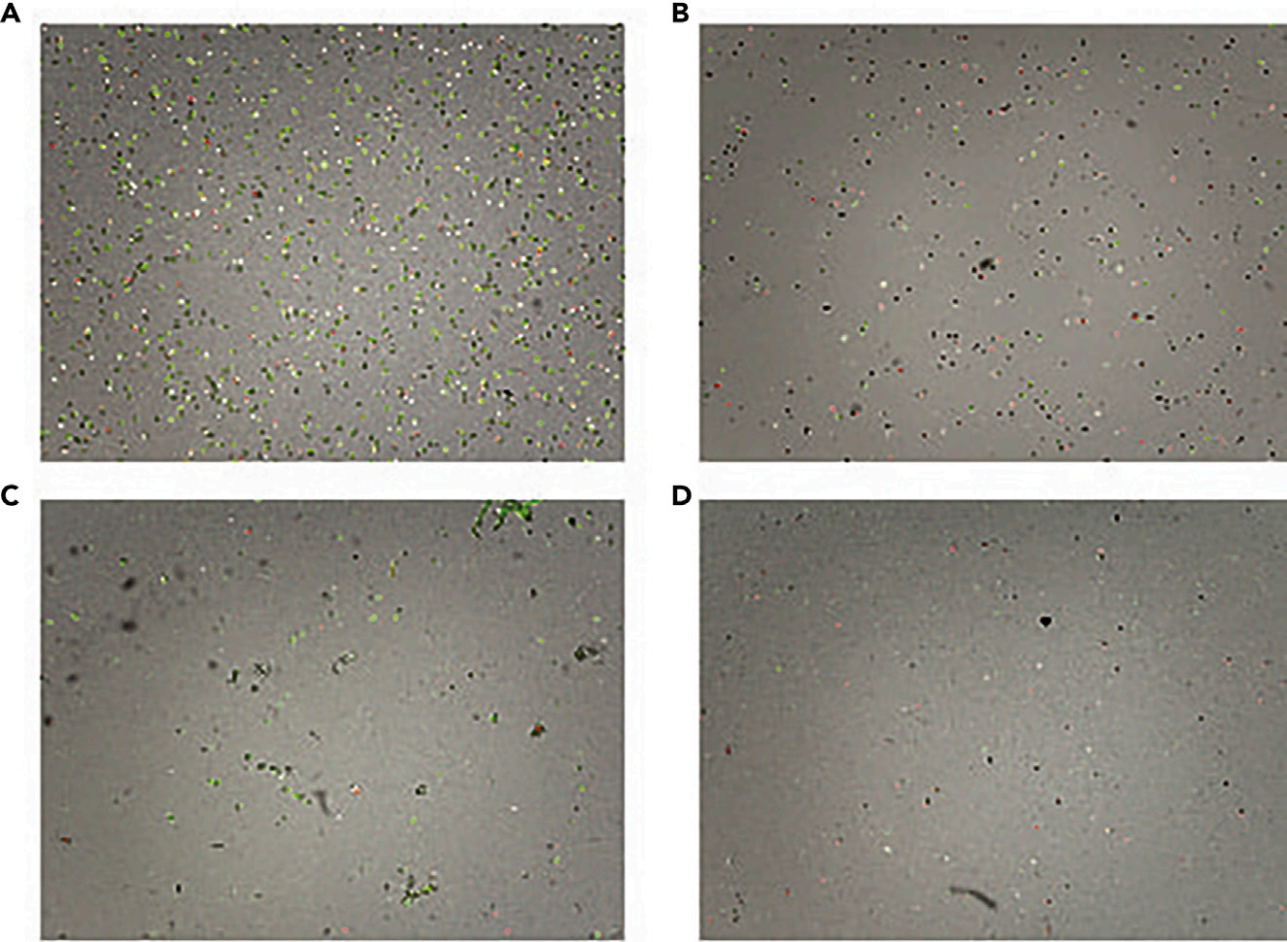
Examples of the good, suboptimal quality and clear dropouts from fine needle aspirate samples during preprocessing prior to single cell droplet generation (A) FNA_Sample1 – Good quality sample, good cell concentration, good viability, clear single cell suspension. Total cell concentration: 3.78 × 10^6^ cells/mL; Live cell concentration: 3.10 × 10^6^ cells/mL; Dead cell concentration: 6.76 × 10^5^ cells/mL; Viability: 82.1%. (B) FNA_Sample2 – Suboptimal sample: good cell concentration, but poor viability, clear single cell suspension. Total cell concentration: 1.07 × 10^6^ cells/mL; Live cell concentration: 5.55 × 10^5^ cells/mL; Dead cell concentration: 5.11 × 10^5^ cells/mL; Viability: 52.1%. (C) FNA_Sample3 – Suboptimal sample: acceptable cell concentration, good viability, visible fibers and cell clumps in single cell suspension. Total cell concentration: 4.15 × 10^5^ cells/mL; Live cell concentration: 3.42 × 10^5^ cells/mL; Dead cell concentration: 7.24 × 10^4^ cells/mL; Viability: 82.5%. (D) FNA_Sample4 – Clear drop out: low cell concentration, poor viability. Total cell concentration: 2.01 × 10^5^ cells/mL; Live cell concentration: 4.02 × 10^4^ cells/mL; Dead cell concentration: 1.61 × 10^5^ cells/mL; Viability: 20.0%. All image magnifications: 1.4×, taken by Luna cell counter. Labeled in green alive cells, in red dead cells.
  b. It is highly recommended to filter the cell suspension through a 35 μM cell strainer. E.g., FACS tubes with blue filter caps. If debris and clumps are present (see Figure 3C, FNA_Sample3 for debris and fibers present in single cell suspension), more than one filtering step might be required to achieve a fine single cell suspension.
    **CRITICAL:** Wash the filter cap with 2−3 mL of PBS to reduce cell loss during the filtering step. Usually 10–30% of cell loss is expected.
  c. Transfer the filtered cell suspension into a new 5 mL Eppendorf tube (to reduce carryover of debris and fibers) and spin down at 300 × g for 5 min at 4°C.
  d. Resuspend cells in 50–500 μL of PBS with 0.04% BSA and count with AOPI live/dead staining. For single cell droplet generation the cell concentration was brought to 700–1200 cells/μL. Good example shown in Figure 3A, FNA_Sample1, good cell concentration, good viability, clear single cell suspension.

5. Thawing the frozen FNA sample (work in the BSL2 hood for human primary material). When processing freshly collected FNA sample go directly to step 7.
  a. Prepare a 5 mL Eppendorf or 15 mL centrifuge tube and put 1 mL of cold PBS (Ca^2+^/Mg^2+^ free) with 0.04% BSA. A 5 mL Eppendorf allows better pellet visualization when the cellular content in the sample is low.
  b. Take frozen cryovials with FNA samples out of the freezer/dry icebox and submerge (not fully; only until the cup line) into a water bath pre-warmed to 37°C. Keep in the water bath until almost thawed. When a small frozen part (around 20% of the total volume) is left, take the cryovial with almost thawed FNA out, open, gently transfer the thawed FNA samples into a 5 mL Eppendorf or 15 mL centrifuge tube, and slowly pipette up and down several times.
  c. Add 1 mL of PBS with 0.04% BSA onto a remaining frozen FNA sample and pipette up and down. This should melt what remains of the FNA sample.
  d. Transfer the rest of the FNA sample into a 5 mL Eppendorf or 15 mL centrifuge tube and bring the total volume to 10 mL with PBS with 0.04% BSA. Do not use big serological pipettes as this might result in loss of the cells on the inner surface of the pipette.
  e. Leave on ice for 10 min. This should allow the remaining DMSO to diffuse from the cells.
  f. Spin down at 300 × g for 5 min at 4°C, remove the supernatant and re-suspend in 100–500 μL of PBS with 0.04% BSA. The resuspension volume depends on the pellet size.

6. Counting cells and viability
  a. Take an appropriate volume of the acridine orange propidium iodide stain (AOPI, e.g., Logos Biosystems Inc., cat.#F23001) mix with cells, load on the counting slide (Photon slides, Ultra-low fluorescence counting slides, cat.# L12005) and count on the available live/dead counting machine (e.g., Luna-FL^TM^ Dual Fluorescence Cell counter). E.g., To save cellular content we used 5 μL of AOPI stain, 5 μL of single cell suspension, and set the cell counter to 1:2 dilution.
    **CRITICAL:** We highly recommend using live-dead cell staining; our best practice was AOPI as it allows quick viability and cell count determination. Check the sample images for clumps and debris. At least one filtering step is needed to obtain a single cell suspension free of debris. Sometimes it might improve the viability and cell number accuracy as the cell counter might count debris as cells (See Figure 3 for optimal, suboptimal and dropout examples images).
    ***Alternatives:*** e.g., Cellometer K2 Fluorescent (Nexcelom) or any cell counter that supports AOPI dual-fluorescence imaging.

7. Processing cells for single cell droplet generation and sequencing library construction should be done according to the 10× protocol for Chromium Next GEM Single Cell 3ʹ Reagent Kits v3.1 or Chromium Single Cell V(D)J Reagent Kits User Guide.
  **Pause point:** After the single cell droplet generation, the sample is processed for a reverse transcription reaction according to the 10× Genomics protocol and could be stored for 72 h at 4°C. See the 10× Genomics protocol for more possible pause points.
  ***Optional steps:***
  a. Dead cell removal: If the viability is below 50% and cell number allows (>0.5 × 10^5^), we recommend performing dead cell removal, following manufacturer instructions, using the EasySep™ Dead Cell Removal (Annexin V) kit (StemCell Technologies, Cat#17899) and EasySep^TM^ Magnet (StemCell Technologies, cat. #18000) designed to deplete apoptotic (Annexin V+) cells by immunomagnetic negative selection. Manufacturer instructions: https://cdn.stemcell.com/media/files/pis/DX21956-PIS_1_0_1.pdf?_ga=2.34218465.1547447083.1547219505%E2%80%93776976877.1534951026. To avoid complete loss of sample, we recommend putting aside 10^4^ cells and performing dead cell removal on the rest of the sample.
  b. Slow spin to remove dead cells and cell debris: If cell number is below 0.5 × 10^5^ a slow spin at 250 × g for 10 min could be performed to increase the viability. After the slow spin, it is important to check for cell pellet presence before discarding the supernatant.
  c. Red blood cell lysis: When the pellet appears red, a simple quick red blood lysis step could be performed using a red blood cell lysis buffer (RBCL, ROCHE, cat.#11814389001). Spin down the cell suspension at 300 × g for 5 min. Resuspend in 500 μL of RBCL buffer and shake at around 22°C for 3–5 min. Top up with PBS with 0.04% BSA (to 5 mL in 5 mL Eppendorf or 10 mL in 15 mL centrifuge tubes) and spin down at 300 × g for 5 min. If the pellet still appears red, repeat the step up to 3 times.

## Expected outcomes

With the FNA procedure, we typically obtained minimum 7000 cells, maximum 0.5 × 10^6^ cells, with average of 5 × 10^4^ cells. The FNA material that we processed for single cell sequencing was between 50%–80% and we used dead cell removal (optional step a) or slow spinning (optional step b) to increase the viability when below 70%. Please see Figure 3 for optimal and suboptimal sample quality examples.

## Quantification and statistical analysis

• Preprocessing
  We recommend using the 10X cellranger (https://support.10xgenomics.com/single-cell-vdj/software/downloads/latest) pipeline for processing the data. The software takes raw sequencing files as input and tags the reads according to barcodes and indices. The command ‘cellranger count’ generates a cell-gene count matrix, whereas the command ‘cellranger vdj’ assembles immune receptor contigs and annotates them.
• Quality control
  The cellranger pipeline also outputs a summary HTML file that should be inspected before proceeding with the downstream analysis. The report may implicate concerns with the library preparation or with the sample quality. This report can be used as a feedback to optimize the library preparation to achieve the desired sequencing depth.
• Downstream analysis
  a. The downstream analysis of the samples may vary greatly depending on the setup of the experiment. In the following, we describe procedures that we view as essential when working with such data. For more details and the related code, we refer to the following GitHub page: https://github.com/uzh-dqbm-cmi/lymphoma_tvec_study .
  b. The ‘cellranger count’ command outputs filtered count matrices in files named barcodes.tsv.gz, features.tsv.gz, matrix.mtx.gz. These files can be read, using the Seurat R package (Butler et al., 2018). This package can be used to filter cells (e.g., by read count, gene count or mitochondrial read ratio), to perform differential gene expression and to visualize the results.
  c. For cell-typing, we recommend using the SingleR R package (Aran et al., 2019).
  d. From the outputs of the ‘cellranger vdj’ command, the filtered_contig_annotations.csv file contains the annotated immune receptor contigs. This file can be merged with the metadata dataframe of the Seurat object to integrate gene expression analysis with immune receptor profiling.
  e. For gene set enrichment and pathway analysis, we recommend using the ClusterProfiler R package (Yu et al., 2012).

## Troubleshooting

### Problem

If the syringe hub does not fill up during the FNA procedure (steps 1d–f)

### Potential solution

Inspect the needle for a clog and repeat the process.

### Problem

If a syringe was accidentally withdrawn from the lesion before the syringe hub fills with tissue material (steps 1c–e).

### Potential solution

Carefully remove the needle from the lesion, release the air from the syringe, and restart the FNA procedure after reattaching the needle.

### Problem

If the lesion appears flat (step 1e)

### Potential solution

In flat lesions, the moving of the syringe must usually be repeated more times before tissue material appears in the syringe hub, compared to thicker or nodular lesions.

### Problem

If tissue is not visible in the syringe hub (step 1)

### Potential solution

If the content of syringe hub is difficult to assess for tissue material, after releasing the content into a vial/cryovial, a drop of material can be taken for visual inspection under the microscope. Minimum viable cell number that we recovered from FNA was around 7 × 10^3^ cells per FNA, which was rather on a low end.

### Problem

Low cell concentration, total cell number less than 10^4^ cells are recovered (step 6)

### Potential solution

Spin down the cell suspension at 350 × g for 5 min at 4°C, remove the excess supernatant and leave around 15 μL of resuspension buffer to achieve a minimum concentration of 700 cells/μL.

### Problem

Suboptimal viability (less than 60%) and the cell number is less than 10^5^ (step 6, Figure 3B)

### Potential solution

Try to perform slow spin at 250 × g for 10 min. Warning: Might result in a drop out or suboptimal quality data after at the data analysis step.

### Problem

Cell number is more than 5 × 10^4^, good viability (more than 80%), but cell suspencion has visible fibers and cell clumps (step 6, Figure 3C).

### Potential solution

Perform additional filtering steps using 35–40 μM cell strainers to remove fibers. If debris and fibers are not removed might result in clogging during single cell droplet generation.

### Problem

The sample has a high median read/cell ratio with a low cell count or a substantially lower cell count than expected (see Downstream analysis).

### Potential Solution

The problem is likely due to the low sample quality or with the microfluidics process. Presumably, too few surviving cells were loaded to the microfluidics device. If some samples have very different coverages that likely leads to batch effects in the downstream analysis. It is recommended to repeat the protocol from the “Preparation of FNA sample for single cell processing” chapter. Additional examples that resulted in drop out at the data analysis step and would need to be re-processed are listed in Table 1.

**Table 1:** Summary of drop-outs and samples with suboptimal quality at data analysis step, AOPI - acridine orange propidium iodide; QC - quality control; BCR - B-cell receptor; TCR - T-cell receptor.

Description of a problem at the data analysis step	Sample ID	Sample preprocessing QC	CellRanger QC output and *interpretation*	Cause of the problem
Samples that were classified as clear drop- outs	Drop-out Sample 1	no AOPI QC of cells (as described in step 7 of the protocol)	Number of Cells: 93 Reads per cell: 809,921 Genes per cell: 66 *Low cell number, few genes per cell*	No proper sample preprocessing QC was performed
	Drop-out Sample 2	no AOPI QC of cells (as described in step 7 of the protocol)	Number of cells: 6,326 Reads per cell: 26,021 Genes per cell: 526 *Low fraction of reads in cells (26.1% , Ideal > 70%)*	No proper sample preprocessing QC was performed
	Drop-out Sample 3	Viability 53.4% Total cell number ~1000 cells	Number of Cells: 64 Reads per Cell: 403,052 Genes per Cell: 72 *Low cell number, few genes per cell*	Low viability Low total cell number
Sample that were classified as drop-outs at immune receptor profiling QC	Drop-out Sample 5	Viability 66.4%	BCR analysis: Cells with productive V-J spanning pair: 1,663 B-cells in sample: 833 TCR analysis: Cells with productive V-J spanning pair: 852 T-cells in the sample: 243 *More BCRs and TCRs than B-cells and T-cells*	Acceptable viability. Probable technical issue during sample processing, overamplification of V(D)J libraries
	Drop-out Sample 6	Viability 40.4%	BCR analysis: Cells with productive V-J spanning pair: 393 B-cells in sample: 773 TCR analysis: Cells with productive V-J spanning pair: 144 T-cells in the sample: 1,853 *High number of B- and T-cells without immune receptor*	Low viability

### Problem

The count matrix in the Seurat object is not in the same order as the cell metadata is (see Downstream analysis).

### Potential Solution

This can be a consequence of merging another dataframe with the metadata dataframe. The Seurat object usually stores the count matrices sorted alphabetically. Sorting the rows of the metadata dataframe by the cell identifiers is likely to solve the problem.

## Limitations

Main limitations for FNA sampling and pre-processing for single cell analysis are necrotic or flat lesions, as cell number and the cell viability obtained from such lesions often result in drop-outs. We have observed suboptimal samples with low cell concentrations, low total numbers, and poor viability (Figure 3D); therefore, we would recommend bringing the viability of cells whenever possible to at least to 70% and concentrating cells to minimum of 700 cells/μL for single cell droplet generation. Lower concentrated cells and viabilities resulted in drop-outs or issues at the data analysis step.

## Resource availability

### Lead contact

Further information and requests for resources and reagents should be directed to and will be fulfilled by the lead contact, Prof. Mitch Levesque mitchell.levesque@usz.ch

### Materials availability

No new material was generated using this protocol.

### Data and code availability

The code used for the analysis of scRNA sequencing data can be found online at https://github.com/uzh-dqbm-cmi/lymphoma_tvec_study .

## Ethical statement

Informed consent was obtained for all human primary material (BASEC# 2018-00379).
